# Mostly harmless? Clinical practice guidelines need further consideration of psychotherapy adverse effects

**DOI:** 10.1177/10398562241282736

**Published:** 2024-09-07

**Authors:** Stephen Allison, Jeffrey CL Looi, Steve Kisely, Tarun Bastiampillai

**Affiliations:** College of Medicine and Public Health, 1065 Flinders University, Adelaide, SA, Australia; and Consortium of Australian-Academic Psychiatrists for Independent Policy and Research Analysis (CAPIPRA), Canberra, ACT, Australia; Academic Unit of Psychiatry and Addiction Medicine, 2219 The Australian National University School of Medicine and Psychology, Canberra Hospital, Canberra, ACT, Australia; and Consortium of Australian-Academic Psychiatrists for Independent Policy and Research Analysis (CAPIPRA), Canberra, ACT, Australia; School of Medicine, 1974 The University of Queensland, Princess Alexandra Hospital, Brisbane, QLD, Australia; and Consortium of Australian-Academic Psychiatrists for Independent Policy and Research Analysis (CAPIPRA), Canberra, ACT, Australia; Department of Psychiatry, 2541 Monash University, Clayton, VIC, Australia; College of Medicine and Public Health, 1065 Flinders University, Adelaide, SA, Australia; and Consortium of Australian-Academic Psychiatrists for Independent Policy and Research Analysis (CAPIPRA), Canberra, ACT, Australia

**Keywords:** adverse effects, psychotherapy, clinical practice guidelines, depressive disorders

## Abstract

The Royal Australian and New Zealand College of Psychiatrists clinical practice guidelines on mood disorders recommend psychotherapy as foundational care for patients with acute depression with minimal discussion of any potential adverse effects. Randomised controlled trial evidence on psychotherapy adverse effects is limited. This is problematic because clinicians must balance the benefits of treatment against the harms, and clinical decisions become skewed without data on adverse effects. We suggest that clinical practice guidelines should be more guarded about recommending psychotherapy and add consensus statements on adverse effects for informed consent and clinical decision-making.

*Regarding the description of the Earth in the Hitchhiker’s Guide to the Galaxy:*

‘Oh yes, well I managed to transmit a new entry off to the editor. He had to trim it a bit, but it’s still an improvement’.

‘And what does it say now?’ asked Arthur.

‘Mostly harmless*’.*

*Do* *uglas Adams, The Complete Hitchhiker’s Guide to the Galaxy: The Trilogy of Five*

The benefits of psychotherapy have been well established but there has been much less focus on potential adverse effects. For example, six types of psychotherapy are recommended as foundational care for patients with acute major depressive disorders in the Royal Australian and New Zealand College of Psychiatrists clinical practice guidelines on mood disorders, based on each type of psychotherapy being tested in at least 10 randomised controlled trials (RCTs).^1^ However, these guidelines have minimal discussion of psychotherapy adverse effects.

## Positioning treatments according to adverse effects

While antidepressant medication and psychotherapy have similar efficacies for acute major depressive disorders, there are differences in the research on their adverse effects.^1^ Reporting of pharmacotherapy adverse effects is mandatory due to regulatory requirements, so the RANZCP clinical practice guidelines describe antidepressant adverse effects, the majority of which are mild and transient but there is evidence of severe problems such as long-term sexual symptoms and potentially increased youth suicidal behaviour.^1^ Antidepressant withdrawal can also be debilitating.^1^ There remains a lack of RCT-level evidence for the benefits and adverse effects of antidepressants in a wide range of situations including extended use.^1^

In contrast, the reporting of adverse effects is not mandatory for psychotherapy RCTs so most trials do not systematically measure and report them. Hence the risks that patients may experience either mild or serious psychotherapy adverse effects are largely unknown. In the absence of RCT-level data, clinical practice guidelines tend to regard psychotherapy adverse effects as relatively benign, or to borrow from Douglas Adams, ‘mostly harmless’.^1^

However, clinicians have long recognised that psychotherapy, like any therapeutic intervention, can and does have adverse effects. Even empirically supported psychotherapies such as cognitive behavioural therapy (CBT) and psychodynamic therapy that have been shown to be beneficial for acute major depression in multiple RCTs have adverse effects. In a ground-breaking article on psychotherapy adverse effects, Berk and Parker describe how CBT can intensify rumination and introspection, which increases depression particularly amongst those with obsessive personalities.^2^ They also suggest that long-term psychodynamic therapy may promote regression and withdrawal effects amongst some patients with dependency issues, which can generate transitory deterioration necessitating specialist management as noted by the RANZCP clinical practice guidelines on mood disoders.^1–3^ Future RCTs should measure the specific adverse effects of each of the six empirically supported psychotherapies for acute depressive disorders.^1^

## Defining psychotherapy adverse effects

The World Health Organization defines adverse effects as negative consequences of treatment that are subjectively unpleasant and associated with suffering (physical, psychological, or social) or impairment in functioning.^4^ Psychotherapy adverse effects are negative consequences of a correctly applied psychotherapy that are reported by the patient, close family members and/or the treating therapist (Box).^5^ These adverse effects can be differentiated from the negative impact of incorrect treatment, malpractice or boundary violations. The patient’s lived experience of psychotherapy is the most important aspect. Patients describe clinical deterioration, increased emotional lability, new symptoms, re-emerging traumatic memories, or demoralisation due to psychotherapy.^2,6,7^ Patients often attribute adverse effects to therapist behaviour, hindering aspects of the therapeutic relationship, poor treatment fit, or direct negative impacts of the type of psychotherapy.^7^ Clinical experience suggests that adverse effects are more likely when the patient becomes overly dependent on the therapist in intensive therapy, adopts the sick role, and loses the capacity to independently problem-solve.^2^

**Box.** Defining psychotherapy adverse effects.

Adverse effects: All negative reactions to an appropriately delivered psychotherapy as reported by the patient, or by a significant other/close family member, or as observed by the treating therapist.
Serious adverse events: Nonlife-threatening events (e.g. hospitalisation, significant deterioration of symptoms), or life-threatening events including suicidality, death, or risk to others (e.g. threat, neglect, or abuse) that occur during psychotherapy
Research attribution: Independent researchers’ judgements whether the negative outcomes observed in a trial of psychotherapy are either unrelated, unlikely to be related, possibly related or likely to be caused by the psychotherapeutic intervention.
Adapted from Klatte R, Strauss B, Flückiger C, et al. Adverse events in psychotherapy randomized controlled trials: A systematic review. *Psychother Res* 2023; 13: 1-16.

A Royal College of Psychiatrists-linked survey of more than 14,000 National Health Service (NHS) patients in England found 5% experienced lasting adverse effects from psychotherapy.^8^ People who were lesbian, gay or bisexual and ethnic minorities, were more likely to report adverse effects, while people aged over 65 were less likely report them.^8^ A more recent NHS survey revealed that 14% of patients reported lasting adverse effects from psychotherapy for depression.^9^ Adverse effects were more likely if patients described not receiving the right number of sessions and not discussing progress with their therapist.

Serious adverse events may also occur during psychotherapy including emergency care, hospitalisation, self-harm, suicidality, completed suicide, or increased risk to others (e.g. threat, neglect, or abuse).^5^ Whether psychotherapy reduces the risk of serious adverse events or whether psychotherapy precipitates serious adverse events in some instances has not been studied systematically.^5^ Impossibly large RCTs would be needed to examine rare events such as suicide.

## Clinical practice guidelines and psychotherapy adverse effects

There is now a large amount of evidence demonstrating that psychotherapy is effective for acute major depressive disorders although a recent umbrella review published in World Psychiatry found that effect sizes are generally small (<0.50).^10^ Only 1% to 17% of the trials of psychotherapy for depressive disorders have low risk of bias and if meta-analyses take risk of bias into account, effect sizes decrease.^10^ Approximately 43% of adults achieve remission with psychotherapy for depression with no significant differences between the various psychotherapies, compared to 23% in passive control groups, and 33% in care-as-usual control groups.^10^ So overall, evidence-based psychotherapy is effective for 10%–20% of adults treated for depression.

A similarly neat empirical summary cannot be provided for psychotherapy adverse effects. The American Psychological Association clinical practice guidelines on depression support the efficacy of psychotherapy but rate the evidence on adverse events as insufficient/very low.^11^ Psychotherapy trial reporting of adverse effects falls below the recommended gold-standard, which comprises comprehensive measurement during treatment and follow-up.^5^ RCTs should use standardised questionnaires and when necessary semi-structured interviews to measure psychotherapy adverse effects at various time points. Independent assessors of adverse effects are helpful because of potential allegiance bias amongst psychotherapy researchers. Finally, RCTs require additional resources to record and report psychotherapy adverse effects.

This insufficient/very low level of evidence on psychotherapy adverse effects creates difficulties for clinicians.^11^ Clinicians must be able to balance the benefits of treatment against harms for each patient, and clinical decisions become skewed without data on adverse effects. The process of informed consent is compromised because it is not possible to provide patients with a summary of the empirically based evidence on the adverse effects associated with a particular type of psychotherapy. While six psychotherapies have been shown to be effective for acute depressive disorders in at least 10 RCTs,^1^ the number of trials with comprehensive measurement and reporting of adverse effects is considerably fewer.^5,12^ This means that clinical practice guidelines cannot weigh the benefits of psychotherapy against potential harms and the strength of treatment recommendations is reduced accordingly.^11^ The lack of RCT-level evidence on psychotherapy adverse effects undermines the positioning of psychotherapy as foundational care for patients with acute depressive disoders.^1^ Thus, clinical practice guidelines should be more guarded on psychotherapy treatment recommendations because the empirical study of the safety of psychotherapy is limited but survey results indicate that many patients report lasting adverse effects.^8,9^

Where RCT-level evidence is unavailable, clinical practice guidelines can include consensus statements.^1^ We suggest that consensus statements on psychotherapy adverse effects could improve the clinical utility of clinical practice guidelines on depression. These statements should be co-developed by patients with lived experience of psychotherapy adverse effects, clinicians with practical knowledge of psychotherapeutic assessment and management, and importantly, independent scientific researchers who are not biased toward or against psychotherapy. These consensus statements would be useful for psychiatry training on informed consent and shared decision-making about psychotherapy in clinical practice.

These suggestions are based on our interpretation of recent systematic reviews on the measurement and reporting of adverse effects in psychotherapy trial protocols and the published reports of psychotherapy RCTs.^5,12^ We have not undertaken an independent critical and systematic review. Based on these published reviews, there has not been rapid and substantial progress in the 15 years since Berk and Parker drew attention to psychotherapy adverse effects.^2^ Clinical practice guidelines should emphasise this fact and raise standards so that the practice of comprehensively measuring psychotherapy efficacy but not adverse effects is no longer acceptable on the grounds of patient safety.
